# Massive sympathetic nerve infiltration in advanced hepatic alveolar echinococcosis: a case report and review of the literature

**DOI:** 10.1186/s12879-022-07470-8

**Published:** 2022-05-23

**Authors:** Zongding Wang, Tiemin Jiang, Tuerganaili Aji, Hao Wen

**Affiliations:** 1 State Key Laboratory of Pathogenesis, Prevention, and Treatment of High Incidence Diseases in Central Asia, The First Affiliated Hospital of Xinjiang Medical University, NO.137 Li Yu Mountain South Road, Ürümqi, 830054 Xinjiang People’s Republic of China; 2 Hepatobiliary and Hydatid Disease Department, First Affiliated Hospital of Xinjiang Medical University, NO.137 Li Yu Mountain South Road, Ürümqi, 830054 Xinjiang People’s Republic of China

**Keywords:** Alveolar echinococcosis, Sympathetic nerve, Neural infiltration, Liver transplantation, Case report

## Abstract

**Background:**

Alveolar echinococcosis is a zoonotic disease that mostly affects the liver, with vascular invasion and a protean clinical symptom. However, no reports of sympathetic nerve infiltration in hepatic alveolar echinococcosis have been reported. Here, we report a case of hepatic alveolar echinococcosis in a 33-year-old man. In this end-stage case, the lesion was heavily involved in the large vessels and biliary tract, and immunohistochemistry also incidentally revealed extensive nerve infiltration in the specimens after surgical treatment. Subsequently, neural classification was identified.

**Case presentation:**

We herein report a case of advanced hepatic alveolar echinococcosis with macrovascular invasion and sympathetic nerve infiltration. In this case, inferior vena cava (IVC), the portal vein and bile duct were infiltrated. Ultimately, according to our experience, ex vivo liver resection and autotransplantation (ELRA) was the optimal treatment way to perform for this unresectable patient. Samples were collected from normal liver tissue, junction tissue and the lesion. Hematoxylin–eosin (HE) staining was used to confirm the diagnosis. Neural infiltration was observed by immunohistochemical staining with protein gene product 9.5 (PGP9.5). Fluorescence colocalization was determined with PGP9.5 and tyrosine hydroxylase (TH). These results suggest that a large amount of sympathetic nerve infiltration occurred at the junction.

**Conclusion:**

This study suggests that advanced hepatic alveolar echinococcosis shows infiltrating growth, often invades the large vessels and biliary ducts, and may be accompanied by sympathetic nerve infiltration.

## Background

Echinococcosis, a zoonotic disease caused by cestodes of the genus *Echinococcus* (family Taeniidae), remains a major public health issue. Alveolar echinococcosis (AE) is mainly caused by infection and prevalent in humans and animals. Its main pathological feature is the presence of metacestode (larva) of *E. multilocularis* in the internal organs, which destroy normal organ tissue and result in alveolar echinococcosis. The most targeted organ for echinococcosis is the liver (95%) [1]. In fact, exceptionally, some primary extra-hepatic AE localizations may be observed. It results in a mortality rate of 90% after 10 to 15 years if untreated [2]. We report a case of progressive hepatic alveolar echinococcosis who had tumor-like nodule formation and massive sympathetic nerve infiltration.

## Case presentation

The patient was a 33 years-old male who was a herder living in Tibetan Plateau. He denied any history of smoking, alcohol, food allergy and drugs, and had no evident past medical history or history evident in family members. He was admitted to our hospital because of intermittent jaundice and scleral icterus. Also, he complained of bloating and an occasional bout of diarrhea. The patient did not receive any medications prior to admission. Physical examination revealed an epigastric mass and mild pitting edema in both lower limbs.

**Fig. 1:**
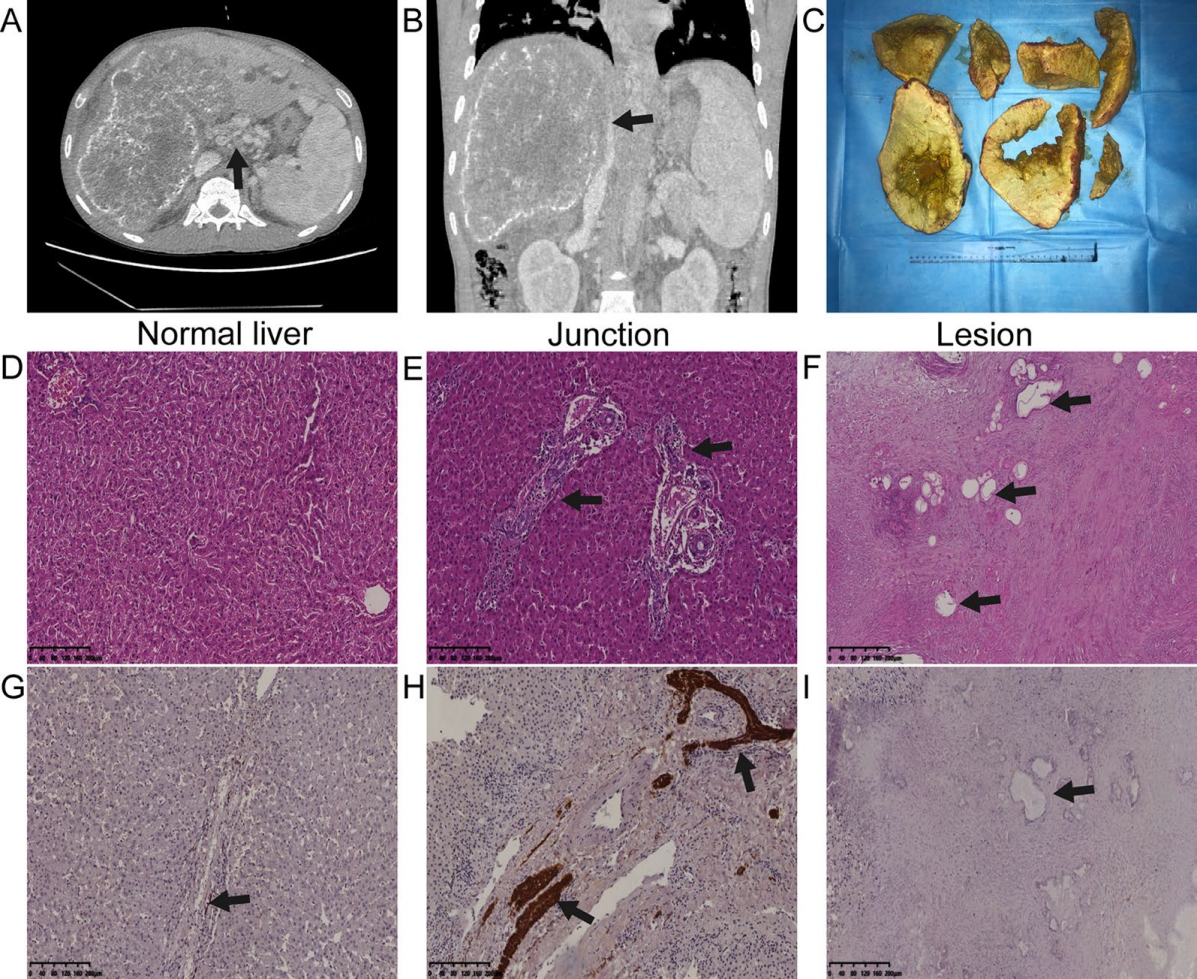
Contrast-enhanced computed tomography (CT) image, resected specimens, HE staining and immunohistochemical staining (scale bar: 200 µm).** A**–**C** Macroscopic aspect of the resected liver. **A** Cross-sectional imaging CT scanning, presenting cavernous transformation of the portal vein (black arrow). **B** Preoperative coronal view of CT scans, with compression of the inferior vena cava (black arrow). **C** Arrows to indicate the necrotic central areas, surrounded by active metacestode area and dense fibrous tissue. **D**–**F** HE staining. **D** Normal appearance of hepatic tissue. **E** Massive inflammatory cell infiltration in junctional tissue. **F** Concentric circular texture and extensive fibrous tissue in the lesion (black arrow shows *E. multilocularis* microcysts). **G**–**I** Immunohistochemical staining of nerve fibers (PGP9.5). **G** Normal liver tissue. **H** Positive expression observed as yellow or brown staining in the junction (black arrow). **I** Few nerve fibers within the metacestode infiltrated area (black arrow.)

Laboratory investigation showed that hemoglobin was 93.0 g/dL, the white blood count was 5.85 × 10^9^/L, the platelet count was 184.0 × 10^9^/L, the international normalized ratio was 1.17, glucose was 4.15 mmol/L, and blood urea nitrogen was 3.28 mmol/L. Liver function tests showed the bilirubin total/direct was 285.70/136.31 µmol/L. Aspartate aminotransferase was 75.70 U/L, alanine aminotransferase was 44.45 U/L, and gamma glutamyl transferase was 79.47 U/L. Alpha-fetoprotein (AFP) was 1.99 ng/mL, CA 125 (cancer antigen 125) was 153.50, and carcinoembryonic antigen (CEA) was 1.69 ng/mL. Contrast-enhanced abdominal CT showed a 19.32 cm × 13.85 cm mass in the right hepatic lobe, encroaching the second hepatic hilum and compressing the inferior vena cava (IVC), and a large number of calcifications were observed. The right portal vein, middle and right hepatic veins and bile duct were all involved. Signs of portal hypertension, such as cavernous transformation of the portal vein (CTPV), ascites, and splenomegaly, were observed (Fig. 1A, B). Due to the low sensitivity and specificity of AE molecular diagnosis, we did not adopt it. On the basis of the long-life history of epidemic area, abdominal CT scan findings and laboratory examinations, a presumptive diagnosis of AE with Budd–Chiari syndrome (BCS) was made. The patient’s staging score was P3N0M0 (stage III a) based on parasitic liver mass, adjacent organ involvement, and metastasis (PNM) [3].

**Fig. 2:**
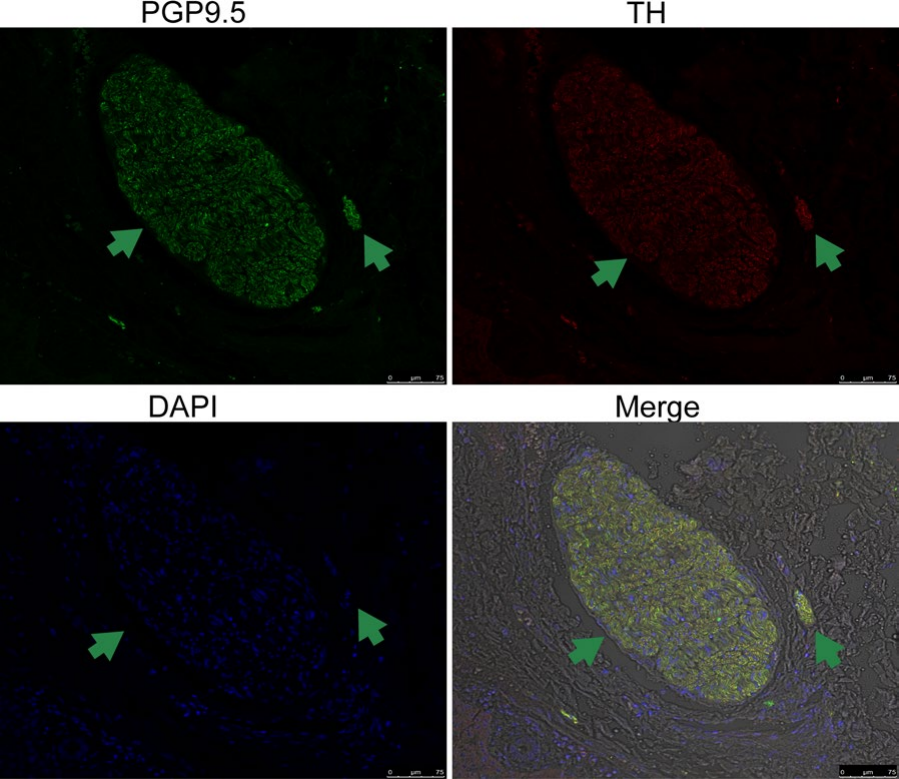
Hepatic sympathetic nerves were identified by confocal laser scanning microscopy. The results of PGP9.5/Tyrosine hydroxylase (TH) colocalization staining showed that hyperplastic sympathetic nerves in junctional tissue. The arrowhead showed the sympathetic nerves. (Scale bar: 75 µm)

Preoperative biliary drainage was performed by endoscopic retrograde cholangiopancreatography (ERCP). The jaundice quickly declined after 3 weeks. Subsequently, emergency endoscopic hemostasis was conducted for upper gastrointestinal bleeding due to portal hypertension. The patient underwent ex vivo liver resection and auto transplantation (ELRA) after the extrahepatic lesions were resected. Samples were taken from the liver lesion, normal tissue, and the junction between each other (Fig. 1C). All liver tissues were fixed for more than 24 h, and then cut into 5 μm sections after paraffin embedding. Hematoxylin–eosin (HE) staining and immunohistochemical staining were performed according to the instructions [4]. HE staining showed abundant granulomatous reaction and coagulative necrosis around the laminated parasitic membranes (Fig. 1D–F). A large number of nerves were observed at the junction by immunohistochemistry (Fig. 1G–I), and these were identified as sympathetic nerves by fluorescence colocalization (Fig. 2). Follow-up 3 months after treatment showed complete relief of the symptoms.

## Discussion and conclusion

AE is a regionally endemic disease [5]. The fecal–oral route is the main mode of transmission of AE. After adult *echinococcus multilocularis* mature in the intestinal tract of the definitive host, it produces a large number of eggs which are released into the environment through the feces of carnivores. Humans are usually indirectly involved in *E. multilocularis* eggs transmission as intermediate hosts. The eggs hatch in the human intestine after being ingested by humans in the contaminated environment, a small amount larvae pass through the intestinal wall into lymphatic vessels or portal vein. It usually settles in the liver and develops as larvae [1]. They also travel through the bloodstream to other organs, including the brain [6, 7], lungs [8, 9], and bone tissue [10, 11].

Liver is the main target organ of *E. multilocularis*. The pathological morphological characteristics are mainly shown as vesicle aggregation, with different sizes. The gross anatomy is often characterized by a large or single nodular, yellowish or white cystic mass with a hard texture. The growth of AE is characterized by strong invasiveness and unclear boundary with surrounding organs and tissues. Lymphatic or vascular invasion provides an opportunity for distant metastasis [12]. The most common site of extrahepatic metastases of AE patients is the lung, followed by brain, bone, and kidney, while heart and other organs are rarely involved. The early stage of AE is asymptomatic and usually lasts 10 to 15 years, which results in losing the best opportunity for early diagnosis and treatment [10].

In terms of treatment, radical surgery often needs to be combined with albendazole medication to achieve the purpose of radical treatment, which is considered as the major treatment for clinically diagnosed AE patients. Surgical procedures include radical hepatectomy, ELRA, classical allogeneic liver transplantation, and palliative treatment (partial resection of the lesion, centro-parasitic abscess or biliary drainages, surgical biliary derivations). Liver transplantation is the main indication for patients with end-stage hepatic alveolar echinococcosis [13–16]. The major advantages of ELRA in patients with advanced AE over classical allogeneic liver transplantation include the absence of organ donors [1, 17] and postoperative immunosuppressive therapy [18, 19]. Therefore, in this case, we reported a patient who lost the opportunity for conventional surgical procedures and underwent ELRA.

The liver is one of the most highly innervated organs in mammals. Autonomic innervation includes vagus and sympathetic fibers, which coordinate with each other to achieve the balance of normal functions. Hepatic nerves are involved in the regulation of various physiological functions, such as carbohydrate metabolism, glucose regulation, lipid metabolism, food intake, and biliary secretion [20]. To date, only one case of hepatic AE has been reported with nerve infiltration, however, the types of nerves remain unclear [21]. Our study addressed this question, we described the location of the nerves, which was identified as sympathetic nerve.

In addition, there is an overwhelming body of evidence that the sympathetic system participates in the so-called “fight and flight response” [22], which is believed to be associated with the inflammatory response [23]. Some studies also confirmed that the sympathetic nervous system promotes hepatocarcinogenesis [24]. Furthermore, the sympathetic nerve is also involved in the metastatic process observed in malignant lesions [25]. Similarly, massive inflammatory cell infiltration in junctional tissue was observed by histopathological staining, which is consistent with the location of sympathetic infiltration. Whether sympathetic nerves are involved in inflammatory reactions in AE needs to be verified by more patient samples. Moreover, the pathological features of AE are similar to those of cancer, and whether the sympathetic nerve plays the same role in AE also needs to be confirmed by further experiments.

In conclusion, this case demonstrates aggressive growth in patients with advanced hepatic alveolar echinococcosis. There is a large amount of sympathetic infiltration in liver tissue around the lesion, which may be involved in immune regulation.

## Data Availability

The datasets generated and/or analyzed during the current study are available in the [jianguoyun] repository, [https://www.jianguoyun.com/p/DdwAc78QtJiDChi27poE].

## Abbreviations

AE: Alveolar echinococcosis
IVC: Inferior vena cava
BCS: Budd–Chiari syndrome
ELRA: Ex vivo liver resection and auto transplantation
HE: Hematoxylin–eosin
TH: Tyrosine hydroxylase
PGP9.5: Protein gene product 9.5
cavernous CTPV: Transformation of the portal vein
ERCP: Endoscopic retrograde cholangiopancreatography
